# Rare Occurrence of Lip Spindle Cell Lipoma

**DOI:** 10.1155/2015/382925

**Published:** 2015-02-25

**Authors:** Sandra Girgis, Leo Cheng

**Affiliations:** ^1^Oral and Maxillofacial Head and Neck Surgery, Homerton University Hospital, Homerton Row, London E9 6SR, UK; ^2^Oral and Maxillofacial Head and Neck Surgery, Homerton University Hospital, St. Bartholomew's and the Royal London Hospital, London E9 6SR, UK

## Abstract

Spindle cell lipoma (SCL) is a rare distinct variant of lipoma, which presents as a painless, circumscribed, slow-growing, superficial lesion on the lip and can mimic a minor salivary gland tumour. We present a slow growing lower lip lesion and its management. *Case Report*. A 38-year-old female gave an eight-year history of a slow-growing mass on her lower lip with intermittent change in size. She presented with a submucosal nodule and thin overlying mucosa adjacent to the vermilion border. Surgical excision was carried as the diagnostic and therapeutic approach. *Conclusion*. Lip SCL is rare, and surgical excision is advocated in order to exclude underlying pathology and minor salivary gland tumours.

## 1. Background

Lipomas are uncommon in the oral cavity and are reported to occur in only 1–4% of cases, with no gender predilection [1–4]. They represent 0.5%–5% of all benign oral cavity neoplasms [1]. Cases of oral spindle cell lipoma (SCL) are rare [5], with a relative incidence thought to be approximately 7%, ranging between 2.17 and 9.8% of all oral lipomas.

SCLs are typically seen in the neck, trunk, and shoulder region in elderly males [6–8] in the fifth to seventh decade [9]. They are slow-growing, benign mesenchymal neoplasms, composed of mature adipocytes, usually surrounded by a thin fibrous capsule.

SCLs usually present as painless, well defined [10], circumscribed [1, 3], sessile, pedunculated [11, 12] submucosal or superficial lesion, mainly located at the buccal mucosa [13], ranging from 1 cm [11], but can increase up to 5-6 cm over a period of years [2, 3, 14].

They have excellent prognosis with no recurrence after surgical excision [4, 12, 15–17].

## 2. Case Report

We present a 38-year-old Caucasian female, referred to our oral and maxillofacial unit by her general practitioner, with a history of a slow-growing lesion on her lower lip for approximately eight years. She reported intermittent change in size and bleeding during the winter months.

She was otherwise fit and well with no known allergies.

Socially history revealed she did not smoke and consumed alcohol minimally.

On clinical examination, she presented with a soft tissue mass over the midline of her lower lip, measuring approximately 6 × 6 mm that was a pedunculated, submucosal nodule adjacent to the vermilion border.

After discussion with the patient, she opted to have the lesion excised for cosmetic reasons. An incision along relaxed skin tension line of the lower lip was made, and an encapsulated soft tissue mass was removed with a cuff of adipose tissue.

Differential diagnosis at the time of clinical examination included, mucocele, liposarcoma, and myxoma [18].

### 2.1. Investigations

**Figure 1:**
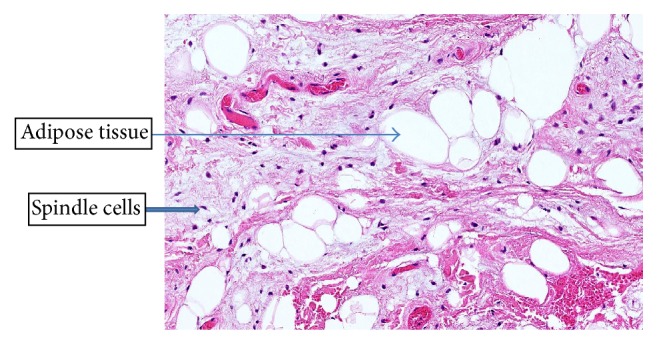
Haematoxylin and eosin staining demonstrating lobules of mature adipose tissue with admixed spindle cells in a fibrous myxoid stroma.

Histological findings revealed evidence of a benign encapsulated tumour, composed of lobules of uniform, mature adipose tissue with admixed spindle cell in a fibrous myxoid stroma. The spindle cells stained positively with CD34 but negatively for the S-100 protein. These features confirmed the lesion was in fact a spindle cell lipoma (Figure 1).

### 2.2. Treatment

We carried out a conservative surgical removal of the lesion under local anaesthetic.

### 2.3. Follow-Up

The patient was reviewed four months after the procedure with no postoperative complications.

## 3. Discussion

SCL first described in 1975 by Enzinger and Harvey [10] is a distinct variant of lipoma derived from prelipoblastic mesenchymal cells [9].

In the English literature, only 35 cases of oral SCLs have been presented to date [19] with an average age range between 23 and 88 years and male predilection [17]. This gender predilection may be partly explained by androgen receptor's reactivity [8]; however, the exact mechanism is still unknown [17].

To our knowledge, only 2 cases of lip SCL have been reported. Billings et al. [6] reported of a 55 yr old female (lower lip, 6 mm), and Manor et al. [3] of a 23 yr old male patient (upper lip, 24 mm).

SCL is a rare entity, particularly on the lip, as such areas are devoid of fat cells. The most common oral site for SCL is the tongue (37%), followed by buccal mucosa (31%) and floor of the mouth (15%). Other sites including the hard palate and alveolar ridge have also been reported.

Aetiology and pathogenesis are still unclear [7, 14, 17]. However, causative factors that have been postulated include trauma, chromosomal abnormality, hereditary, chronic irritation, hormonal imbalance metabolic conditions [7], heredity, fatty generation, and lipoblastic embryonic cell nest in origin [11].

The occurrence of multiple lipomas is associated with Cowden's syndrome or multiple hamartoma syndrome [1]. Multiple head and neck lipomas are observed in neurofibromatosis, Gardener syndrome, encephalocraniocutaneous lipomatosis, multiple familial lipomatosis, and Proteus syndrome [11].

Diagnosis is based on both the clinical and histological characteristics.

Microscopically, SCLs do not differ from normal fat cells; however they do differ metabolically [7] in that they are not used as an energy source as in normal adipose tissue. This may be due to high lipoprotein lipase activity in neoplastic lipoma cells [1].

Intraoral lipomas are described into the following microscopic subtypes: classic lipoma make up 80%, and other variants such as fibrolipoma, intramuscular lipoma, minor salivary gland lipoma (sialolipomas), angiolipoma, and infiltrative and spindle cell lipoma make up the remaining 20%.

Histologically, there are two characteristic features associated with a SCL. First, the lesion is composed of bland mitotically inactive spindle cells arranged in parallel registers between the fat cells, associated with thick rope-like collage bundles [7]. The other is the immunohistochemical stains for CD34 highlighting the bland spindle cell [6] and negative staining with S-100 protein [6, 8]. This was demonstrated in this case.

Another feature of note is the mast cells which are a feature in almost all SCLs [8, 19], also revealed in the histological findings of this case.

The treatment of choice of all histological variants is local surgical excision [7] with good prognosis and rare recurrence [1, 7]. Excision should be carried out with caution to avoid recurrence especially with infiltrating lipomas [2].

This case highlights the fact that SCL should be considered in the differential diagnosis of oral cavity mesenchymal tumours, as well as its malignant counterpart, liposarcoma. It should be noted that although liposarcoma is a common soft tissue neoplasm, its occurrence in the oral cavity is rare [20]. Malignant transformation of SCL has not been reported [16].

Other connective tissue lesions such as granular cell tumour, neurofibroma, and salivary gland lesions (mucocele and mixed tumour) might be included in the differential diagnosis [20].

## 4. Conclusion

SCL, a distinct histological variant of lipoma, is a rare entity in the oral cavity. It is a benign neoplasm, usually asymptomatic and typically slow-growing. Aetiological factors are unclear and definitive diagnosis is confirmed by both clinical presentation and microscopic findings. Treatment is by conservative surgical excision to exclude underlying pathology and minor salivary gland tumours.

## 5. Learning Outcomes

- Intraoral SCL is a rare clinical presentation and usually presents as slow-growing, well circumscribed lesion.
- Aetiology is unclear.
- Accurate diagnosis depends on correct correlation between histological and clinical features.
- SCL has unique histological and immunostaining characteristics.
- Treatment is usually conservative excision with no recurrence and excellent prognosis.
